# Supplementary material for: Structurally and Morphologically Distinct Pathological Tau Assemblies Differentially Affect GVB Accumulation
Source: Int J Mol Sci. 2023 Jun 29;24(13):10865. doi: 10.3390/ijms241310865 (PMC10341458; doi:10.3390/ijms241310865)
Supplement: Supplementary file 1 [file ijms-24-10865-s001.zip › ijms-2441421-supplementary.pdf]

Supplementary information

Structurally and Morphologically Distinct Pathological Tau Assemblies Differentially Affect GVB Accumulation

Marta Jorge-Oliva 1, Jan R. T. van Weering 1,2 and Wiep Scheper 1,2,*

1 Department of Functional Genomics, Center for Neurogenomics and Cognitive Research, Vrije Universiteit Amsterdam, Amsterdam Neuroscience—Neurodegeneration, 1081 HV Amsterdam, The Netherlands; m.jorgeoliva@vu.nl (M.J.-O.); j.vanweering@amsterdamumc.nl (J.R.T.v.W.)

2 Department of Human Genetics, Amsterdam UMC Location Vrije Universiteit, Amsterdam Neuroscience—Neurodegeneration, 1081 HZ Amsterdam, The Netherlands

* Correspondence: w.scheper@amsterdamumc.nl; Tel.: +31-20-5982771

Supplementary Figure S1

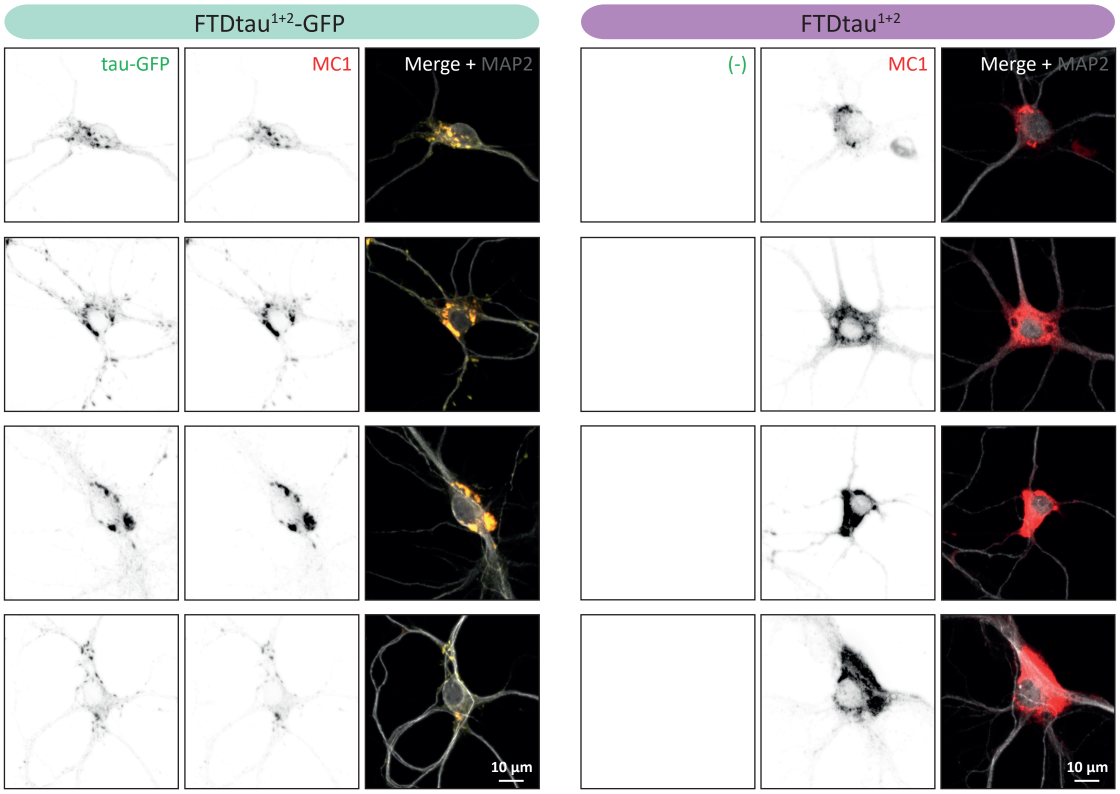

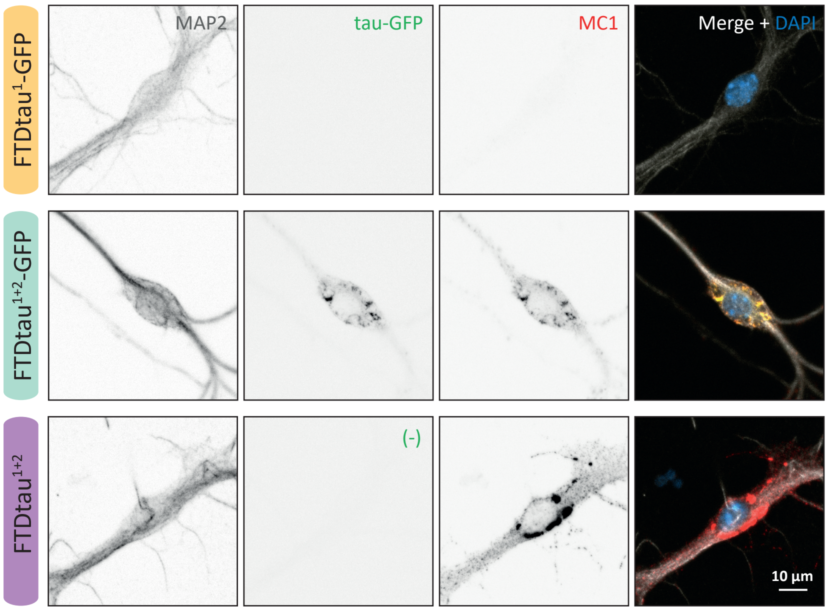

Supplementary Figure 1 Pathological FTDtau^{1+2}-GFP and FTDtau^{1+2} assemblies are MeOH insoluble. **a** Representative confocal images of neurons neurons in the different tau aggregation models as additional examples related to Figure 1b. Neurons are visualised with MAP2 (grey), pathological tau accumulations are marked by MC1 (red) and the green channel corresponds to tau-GFP signal in the case of the FTDtau1+2-GFP model. **b** Representative confocal images of MeOH-fixed neurons transduced with FTDtau^{1+2}-GFP, FTDtau^{1+2} or FTDtau^{1}-GFP as a negative control for aggregation. Immunofluorescence was performed for MAP2 (grey) and MC1 (red); the green channel represents shows tau-GFP signal or its absence (-) in the case of the FTDtau^{1+2} model. DAPI (blue) is also shown in the merged images. Separate channels are shown in greyscale.

Supplementary Figure S2

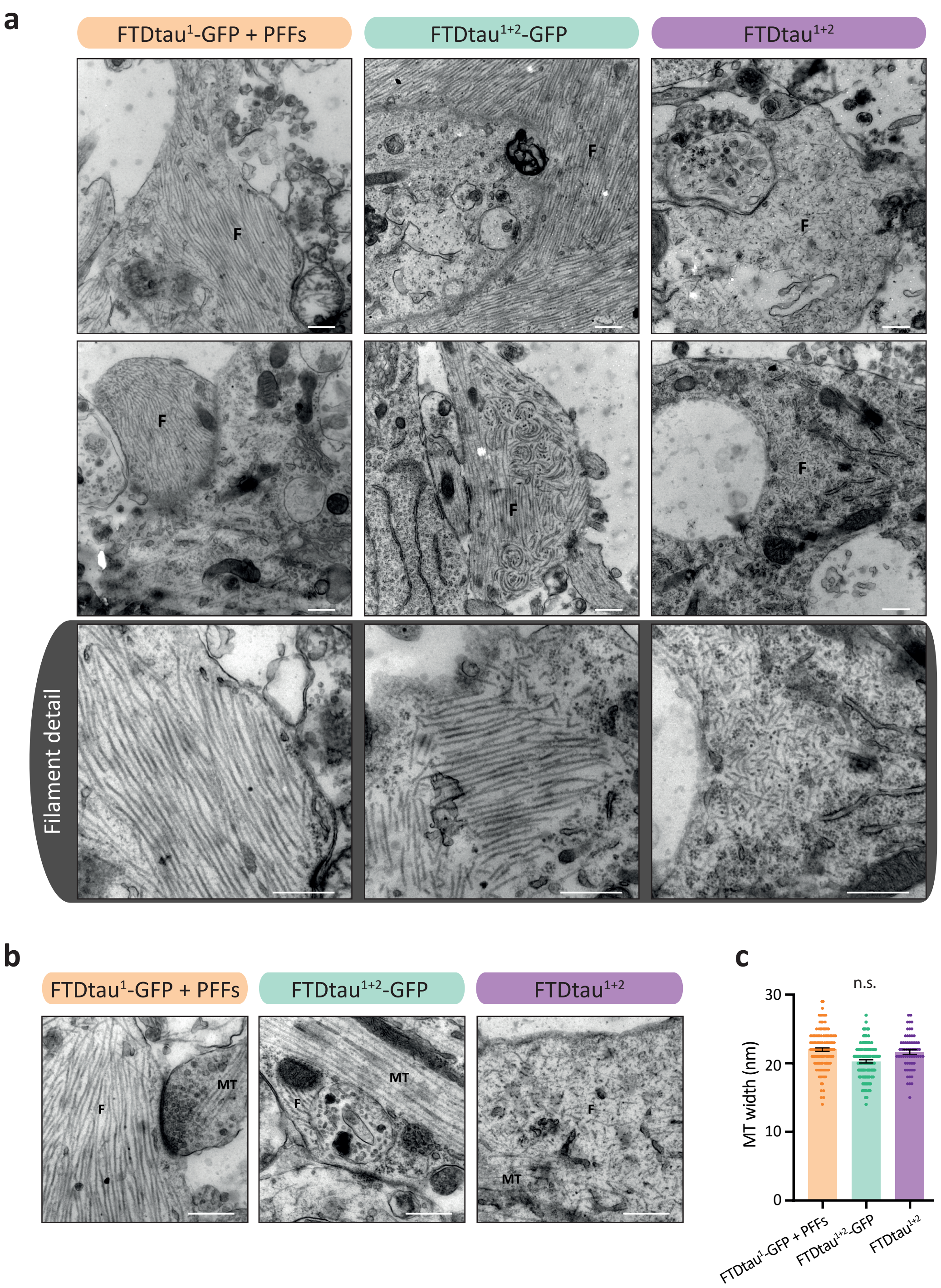

Supplementary Figure 2 Dissimilar ultrastructure of tau filaments but not microtubules in the different tau aggregation models. **a** Representative TEM images of neurons in the different tau aggregation model containing filamentous structures as additional examples to the ones showed in Figure 3a. Tau fibrillar structures are indicated (F). **b**,**c** Analysis of microtubule width in neurons in the different tau aggregation models. **b** Representative TEM images showing microtubular structures (MT) as well as tau filaments (F) in the different models. **c** Quantification of microtubule width. Data points represent single filament values and error bars represent the SEM. N=3 independent experiments. n.s.=not significant, Nested one-way ANOVA with Tukey's post-hoc test. In **a** and **b**, the scale bar represents 500 nm.

Supplementary Table S1: Overview of independent experiments and statistical analysis

Figure	Experiment detail	Number of independent experiments	n employed in analysis (cells if not indicated otherwise)	Statistical test performed	p-value
Fig. 1c	Mean MC1 intensity	3	FTDtau^{1+2}-GFP: 51<br>FTDtau^{1+2}: 60	Nested t-test	p=0.8273, n.s.
Fig. 1d	SD MC1 intensity	3	FTDtau^{1+2}-GFP: 51<br>FTDtau^{1+2}: 60	Nested t-test	p=0.4419, n.s.
Fig. 2c	Tau pathology progression	3	FTDtau^{1+2}-GFP:<br>0 days: 3921<br>1 day: 4111<br>4 days: 4164<br>8 days: 3675<br>11 days: 4097<br>13 days: 4290<br>14 days: 4595<br>15 days: 4180<br><br>FTDtau^{1+2}:<br>0 days: 5566<br>1 day: 5037<br>4 days: 5655<br>8 days: 5515<br>11 days: 5450<br>13 days: 5477<br>14 days: 5775<br>15 days: 5540	Nested t-test per time point	All conditions: p>0.2805, n.s.
Fig. 3b	Filament length	3	(1) FTDtau^{1}-GFP + PFFs: 604<br>(2) FTDtau^{1+2}-GFP: 1063<br>(3) FTDtau^{1+2}: 795<br>n = filaments	Kruskal-Wallis with Dunn's post-hoc test	(1) vs. (2): p<0.0001, ****<br>(1) vs. (3): p<0.0001, ****<br>(2) vs. (3): p<0.0001, ****
Fig. 3c	Filament width	3	FTDtau^{1}-GFP + PFFs: 134<br>FTDtau^{1+2}-GFP: 368<br>FTDtau^{1+2}: 260<br>n = filaments	Kruskal-Wallis with Dunn's post-hoc test	(1) vs. (2): p>0.9999, n.s.<br>(1) vs. (3): p<0.0001, ****<br>(2) vs. (3): p<0.0001, ****
Fig. 3d	Filament angle SD	3	FTDtau^{1}-GFP + PFFs: 23 (10-48; 604)<br>FTDtau^{1+2}-GFP: 30 (18-75; 1063)<br>FTDtau^{1+2}: 23 (20-51; 795)<br>n = micrographs (min-max; total number of filaments)	Kruskal-Wallis with Dunn's post-hoc test	(1) vs. (2): p=0.9289, n.s.<br>(1) vs. (3): p=0042, **<br>(2) vs. (3): p=0.0001, ***
Fig. 4b	Mean MC1 intensity	8	(1) No tau OE: 37490; 44<br>(2) FTDtau^{1+2}-GFP: 25562; 30<br>(3) FTDtau^{1+2}: 28945; 33<br>n = cells; wells/data points	Kruskal-Wallis with Dunn's post-hoc test	(1) vs. (2): p<0.0012, **<br>(1) vs. (3): p<0.0086, **<br>(2) vs. (3): p>0.9999, n.s.
Fig. 4d	GVB load	8	(1) No tau OE: 39637; 44<br>(2) FTDtau^{1+2}-GFP: 28874; 29<br>(3) FTDtau^{1+2}: 28945; 33<br>n = cells; wells/data points	Nested one-way ANOVA with Tukey's post-hoc test	(1) vs. (2): p>0.7154, n.s.<br>(1) vs. (3): p<0.0003, ***<br>(2) vs. (3): p=0.0032, **

Sup. Fig. 2c	Hollow filament width	3	(1) FTDtau^{1}-GFP + PFFs: 139<br>(2) FTDtau^{1+2}-GFP: 119<br>(3) FTDtau^{1+2}: 62<br><br>n = filaments	Nested one-way ANOVA with Tukey's post-hoc test	(1) vs. (2): p=0.0647, n.s.<br>(1) vs. (3): p=0.7805, n.s.<br>(2) vs. (3): p=0.2177, n.s.
